# Supplementary material for: HIVprotI: an integrated web based platform for prediction and design of HIV proteins inhibitors
Source: J Cheminform. 2018 Mar 9;10:12. doi: 10.1186/s13321-018-0266-y (PMC5845081; doi:10.1186/s13321-018-0266-y)
Supplement: Supplementary file 2 — Additional file 2. Source code of HIVProtI web server. [file 13321_2018_266_MOESM2_ESM.zip › HIVprotI_Source-code/hivprotI/weka-3-6-6/doc/weka/classifiers/trees/lmt/ResidualSplit.html]

ResidualSplit


---


|  |  |  |
| --- | --- | --- |
| **Overview** \| **Package** \| **Class** \| **Tree** \| **Deprecated** \| **Index** \| **Help** \| **Weka's home** | |  |
| **PREV CLASS**   NEXT CLASS | **FRAMES**    **NO FRAMES**     **All Classes** |  |
| SUMMARY: NESTED \| FIELD \| CONSTR \| METHOD | DETAIL: FIELD \| CONSTR \| METHOD |  |


---


## weka.classifiers.trees.lmt Class ResidualSplit

```
java.lang.Object
  weka.classifiers.trees.j48.ClassifierSplitModel
      weka.classifiers.trees.lmt.ResidualSplit
```

**All Implemented Interfaces:**: java.io.Serializable, java.lang.Cloneable, RevisionHandler

---

``` public class ResidualSplit extends ClassifierSplitModel ```

Helper class for logistic model trees (weka.classifiers.trees.lmt.LMT) to implement the
splitting criterion based on residuals of the LogitBoost algorithm.

**Version:**
:   $Revision: 1.4 $

**Author:**
:   Niels Landwehr

**See Also:**: Serialized Form

---

**Constructor Summary**

`ResidualSplit(int attIndex)`
  
          Creates a split object


| **Method Summary** | |
| --- | --- |
| `void` | `buildClassifier(Instances data)`             Method not in use |
| `void` | `buildClassifier(Instances data, double[][] dataZs, double[][] dataWs)`             Builds the split. |
| `boolean` | `checkModel(int minNumInstances)`             Checks if there are at least 2 subsets that contain >= minNumInstances. |
| `double` | `entropyGain()`             Computes entropy gain for current split. |
| `java.lang.String` | `getRevision()`             Returns the revision string. |
| `java.lang.String` | `leftSide(Instances data)`             Returns name of splitting attribute (left side of condition). |
| `java.lang.String` | `rightSide(int index, Instances data)`             Prints the condition satisfied by instances in a subset. |
| `java.lang.String` | `sourceExpression(int index, Instances data)`             Method not in use |
| `double[]` | `weights(Instance instance)`             Method not in use |
| `int` | `whichSubset(Instance instance)`             Returns index of subset instance is assigned to. |

**Methods inherited from class weka.classifiers.trees.j48.ClassifierSplitModel**

`checkModel, classifyInstance, classProb, classProbLaplace, clone, codingCost, distribution, dumpLabel, dumpModel, numSubsets, resetDistribution, sourceClass, split`

**Methods inherited from class java.lang.Object**

`equals, getClass, hashCode, notify, notifyAll, toString, wait, wait, wait`

**Constructor Detail**

### ResidualSplit

```
public ResidualSplit(int attIndex)
```

:   Creates a split object

    **Parameters:**: `attIndex` - the index of the attribute to split on


**Method Detail**

### buildClassifier

```
public void buildClassifier(Instances data,
                            double[][] dataZs,
                            double[][] dataWs)
                     throws java.lang.Exception
```

:   Builds the split.
    Needs the Z/W values of LogitBoost for the set of instances.

    :   **Throws:**: `java.lang.Exception`

---


### entropyGain

```
public double entropyGain()
                   throws java.lang.Exception
```

:   Computes entropy gain for current split.

    :   **Throws:**: `java.lang.Exception`

---


### checkModel

```
public boolean checkModel(int minNumInstances)
```

:   Checks if there are at least 2 subsets that contain >= minNumInstances.

---


### leftSide

```
public final java.lang.String leftSide(Instances data)
```

:   Returns name of splitting attribute (left side of condition).

    :   **Specified by:**: `leftSide` in class `ClassifierSplitModel`
    :   **Parameters:**: `data` - the data.

---


### rightSide

```
public final java.lang.String rightSide(int index,
                                        Instances data)
```

:   Prints the condition satisfied by instances in a subset.

    :   **Specified by:**: `rightSide` in class `ClassifierSplitModel`

---


### whichSubset

```
public final int whichSubset(Instance instance)
                      throws java.lang.Exception
```

:   **Description copied from class: `ClassifierSplitModel`**
:   Returns index of subset instance is assigned to.
    Returns -1 if instance is assigned to more than one subset.

    :   **Specified by:**: `whichSubset` in class `ClassifierSplitModel`
    :   **Throws:**: `java.lang.Exception` - if something goes wrong

---


### buildClassifier

```
public void buildClassifier(Instances data)
```

:   Method not in use

    :   **Specified by:**: `buildClassifier` in class `ClassifierSplitModel`

---


### weights

```
public final double[] weights(Instance instance)
```

:   Method not in use

    :   **Specified by:**: `weights` in class `ClassifierSplitModel`

---


### sourceExpression

```
public final java.lang.String sourceExpression(int index,
                                               Instances data)
```

:   Method not in use

    :   **Specified by:**: `sourceExpression` in class `ClassifierSplitModel`

---


### getRevision

```
public java.lang.String getRevision()
```

:   Returns the revision string.

    :   **Returns:**: the revision


---


|  |  |  |
| --- | --- | --- |
| **Overview** \| **Package** \| **Class** \| **Tree** \| **Deprecated** \| **Index** \| **Help** \| **Weka's home** | |  |
| **PREV CLASS**   NEXT CLASS | **FRAMES**    **NO FRAMES**     **All Classes** |  |
| SUMMARY: NESTED \| FIELD \| CONSTR \| METHOD | DETAIL: FIELD \| CONSTR \| METHOD |  |


---
